# Comprehensive molecular etiology analysis of nonsyndromic hearing impairment from typical areas in China

**DOI:** 10.1186/1479-5876-7-79

**Published:** 2009-09-10

**Authors:** Yongyi Yuan, Yiwen You, Deliang Huang, Jinghong Cui, Yong Wang, Qiang Wang, Fei Yu, Dongyang Kang, Huijun Yuan, Dongyi Han, Pu Dai

**Affiliations:** 1 Department of Otolaryngology, PLA General Hospital, Beijing, PR China; 2 Department of Otolaryngology, Affiliated hospital of Nantong University, Nantong, Jiangsu Province, 226001, PR China

## Abstract

**Background:**

Every year, 30,000 babies are born with congenital hearing impairment in China. The molecular etiology of hearing impairment in the Chinese population has not been investigated thoroughly. To provide appropriate genetic testing and counseling to families, we performed a comprehensive investigation of the molecular etiology of nonsyndromic deafness in two typical areas from northern and southern China.

**Methods:**

A total of 284 unrelated school children with hearing loss who attended special education schools in China were enrolled in this study, 134 from Chifeng City in Inner Mongolia and the remaining 150 from Nangtong City in JiangSu Province. Screening was performed for *GJB2*, *GJB3*, *GJB6*, *SLC26A4*, *12S rRNA*, *and tRNA*^*ser*(*UCN*) ^genes in this population. All patients with *SLC26A4 *mutations or variants were subjected to high-resolution temporal bone CT scan to verify the enlarged vestibular aqueduct.

**Results:**

Mutations in the *GJB2 *gene accounted for 18.31% of the patients with nonsyndromic hearing loss, 1555A>G mutation in mitochondrial DNA accounted for 1.76%, and *SLC26A4 *mutations accounted for 13.73%. Almost 50% of the patients with nonsyndromic hearing loss in these typical Chinese areas carried *GJB2 *or *SLC26A4 *mutations. No significant differences in mutation spectrum or prevalence of *GJB2 *and *SLC26A4 *were found between the two areas.

**Conclusion:**

In this Chinese population, 54.93% of cases with hearing loss were related to genetic factors. The *GJB2 *gene accounted for the etiology in about 18.31% of the patients with hearing loss, *SLC26A4 *accounted for about 13.73%, and *mtDNA *1555A>G mutation accounted for 1.76%. Mutations in *GJB3, GJB6*, and *mtDNA tRNA*^*ser*(*UCN*) ^were not common in this Chinese cohort. Conventionally, screening is performed for *GJB2*, *SLC26A4*, and mitochondrial *12S rRNA *in the Chinese deaf population.

## Introduction

Hearing impairment is the most common neurosensory disorder in humans, with an incidence of approximately one in 1000 children worldwide. About 50-60% of these cases have a genetic cause [1]. In China, it has been estimated that 30,000 babies are born with congenital hearing impairment per 20 million live births every year [2]. Although some mutational hotspots involved in inherited hearing impairment, such as *GJB2 *235 delC, *SLC26A4 *IVS7-2A>G, and mitochondrial DNA 1555A>G, have been reported in Chinese deaf populations, the molecular etiology of deafness in Chinese children has not been investigated systematically, and effective genetic evaluation strategies for hearing impairment are not available in most areas of China. China is a large country with a population of 1.3 billion, of which 91% are Han ethnic people. Comprehensive genetic analysis of deaf children in different regions of China should be performed to obtain epidemiological information to provide effective genetic testing and accurate counseling.

The most common molecular defects in nonsyndromic autosomal recessive deafness involve Connexin 26, a gap junction protein encoded by the *GJB2 *gene [3-10]. More than 150 mutations, polymorphisms, and unclassified variants of *GJB2 *have been reported to account for the molecular etiology of about 8-40% of patients with nonsyndromic hearing impairment . However, almost 79% of patients with nonsyndromic hereditary deafness in China do not have mutations in *GJB2 *[11]. Indeed, mutations in other connexin genes, such as *GJB6 *for Cx30 and *GJB3 *for Cx31, have been identified and shown to cause hearing impairment [12,13]. Sequence analysis of the *GJB2 *gene in subjects with autosomal recessive hearing impairment has revealed a puzzling problem in that a high number of patients carry only one mutant allele. Some of these families showed clear evidence of linkage to the DFNB1 locus, which contains two genes, *GJB2 *and *GJB6 *[3,14]. Further analysis demonstrated a deletion truncating the *GJB6 *gene, encoding connexin 30, near *GJB2 *in heterozygous affected subjects [15,16].

*SLC26A4 *also makes appreciable contributions to autosomal recessive nonsyndromic deafness, enlargement of the vestibular aqueduct (EVA), and Pendred syndrome. *SLC26A4 *encodes an anion (chloride/iodide) transporter transmembrane protein, pendrin, which is expressed in the thyroid, kidney, and cochlea [17,18]. DNA sequence analysis identified more than 100 different mutations in *SLC26A4 *[8,19-25]. It was reported that *SLC26A4 *mutations accounted for approximately 5% of all cases of prelingual deafness in East Asia, 5% of cases of recessive deafness in south Asia [26], 3.5% in the UK, and 4% in the Caucasian population with nonsyndromic hearing loss [27].

Although the majority of cases with hereditary hearing loss are caused by nuclear gene defects, it has become clear that mutations in mitochondrial DNA (*mtDNA*) can also cause nonsyndromic hearing loss [28,29]. The best studied of these mutations is the 1555A>G mutation in the mitochondrial *12S rRNA *gene. Another recently identified mutation in the mitochondrial *12S rRNA *gene is the 1494C>T in the conserved stem structure of *12S rRNA *[30]. Other nucleotide changes at positions 961 and 1095 in the *12S rRNA *gene have been shown to be associated with hearing loss, but their pathogenic mechanisms of action in the predisposition of carriers to aminoglycoside toxicity are much less clear [31,32]. Several mutations (7444G>A, 7445A>G, 7472insC, 7510T>C, 7511T>C, and 7512T>C) in the mitochondrial *tRNA*^*ser*(*UCN*) ^gene are also known to cause maternally inherited nonsyndromic hearing loss by disrupting the tRNA structure and function [33-35]. The *mtDNA *1555A>G mutation accounts for a small fraction of patients with nonsyndromic hearing loss, with frequencies between 0.6% and 2.5% among different Caucasian populations [36-40] and higher frequencies in Asian countries (3.43%, 3%, and 5.3% in Chinese, Japanese, and Indonesian cohorts, respectively) [41-43].

In the present study, we performed a comprehensive analysis of 6 prominent deafness-related genes, *GJB2*, *GJB3*, *GJB6*, *SLC26A4*, *mtDNA 12S rRNA*, *and mtDNA tRNA*^*ser*(*UCN*)^, in 284 patients with early-onset, nonsyndromic hearing impairment from unrelated families from two typical Chinese areas, Chifeng City in northern China and Nantong City in southern China, to investigate the molecular etiology in order to provide effective risk assessment and genetic counseling for hearing loss patients and their families in China.

## Materials and methods

### Patients and DNA samples

A total of 284 deaf subjects from unrelated families were included in this study; 134 were from Chifeng Special Education School in Inner Mongolia, and 150 were from Nantong Special Education School in JiangSu Province, China. The Huanghe River is the demarcation line between northern and southern China. Chifeng is a typical city in northern China with a population of 4.61 million, and Nantong is a typical city in southern China with a population of 7.74 million. Chifeng and Nantong are moderate on the population scales in northern and southern China, respectively. Chifeng and Nantong both have long histories of 8000 years and at least 5000 years, respectively. No significant population immigration has occurred over the history of the two cities, and the genetic backgrounds of the respective populations remain relatively intact. The two cities have relatively stable economic development, and the living habits and cultural background of the populations are characteristic of northern and southern China, respectively. This cohort of patients consisted of 158 males and 126 females from 3 to 20 years old with an average age of 12.30 ± 2.70 years. Ethnically, the patients consisted of 243 Han, 31 Mongolian, 7 Man, and 3 Hui Chinese. The study protocol was performed with the approval of the ethnicity committee of the Chinese PLA General Hospital. Informed consent was obtained from all subjects prior to blood sampling. Parents were interviewed with regard to age of onset, family history, mother's health during pregnancy, and patient's clinical history, including infection, possible head or brain injury, and the use of aminoglycoside antibiotics.

All subjects showed moderate to profound bilateral sensorineural hearing impairment on audiograms. Careful medical examinations revealed no clinical features other than hearing impairment. DNA was extracted from the peripheral blood leukocytes of 284 patients with nonsyndromic hearing loss and 200 region- and race-matched controls with normal hearing using a commercially available DNA extraction kit (Watson Biotechnologies Inc, Shanghai, China).

### Mutational analysis

DNA sequence analysis of the *GJB2 *coding region plus approximately 50 bp of the flanking intron regions, mitochondrial *12S rRNA *(nt611 to nt2007), and *tRNA*^*ser*(*UCN*) ^(nt7148 to nt8095) genes were amplified by PCR followed by sequencing using the Big Dye sequencing protocol in all patients. The sequence results were analyzed using an ABI 3100 DNA sequencing machine (Applied Biosystems, Foster City, CA) and ABI 3100 Analysis Software v.3.7 NT, according to manufacturer's protocol. Patients with monoallelic *GJB2 *coding region mutation were further tested for *GJB2 *IVS1+1G>A mutation or defects in exon1 and basal promoter of *GJB2*, *GJB6 *309-kb deletion, and deletion of the whole *GJB6 *coding region. The presence of the 309-kb deletion of *GJB6 *was analyzed by PCR [15,16]. A positive control (provided by Balin Wu, Department of Laboratory Medicine, Children's Hospital Boston and Harvard Medical School, Boston, MA) was used for detection of *GJB6 *gene deletions.

Patients with two *GJB2 *mutant alleles, one dominant mutant allele, or *mtDNA *1555A>G mutation were not analyzed for *SLC26A4 *mutations. The exons of *SLC26A4 *of the remaining 227 patients were sequenced individually starting from the frequently mutated exons until two mutant alleles were identified.

Patients with two *GJB2 *mutant alleles, one dominant mutant allele, *mtDNA *1555A>G mutation, or verified EVA were not analyzed for *GJB3 *mutations. The coding exon of *GJB3 *was sequenced in the remaining 188 patients.

Two hundred controls with normal hearing were sequenced to determine the presence of mutations and polymorphisms in the *GJB2, GJB3*, and *GJB6 *genes and *mtDNA 12S rRNA *and *tRNA*^*ser*(*UCN*)^. In addition, all controls were screened for *SLC26A4 *mutations by DHPLC followed by sequencing analysis.

### CT scan and thyroid examination

Fifty-six of 59 patients with mutations or variants in *SLC26A4 *were examined by temporal bone computed tomography (CT) scan for diagnosis of EVA or inner ear malformation based on a diameter of >1.5 mm at the midpoint between the common crus and the external aperture [28]. To evaluate Pendred syndrome, patients positive for *SLC26A4 *mutations or variants were examined by ultrasound scan of the thyroid and determination of thyroid hormone levels. These procedures were performed at the Second Hospital of Chifeng City, Inner Mongolia and hospitals affiliated with Nantong University, China. As perchlorate discharge testing is not a general clinical practice in China, it was not used in this study.

## Results

Among the 284 cases included in this study, 139 cases had prelingual hearing loss, including 94 congenital cases. Fifty-six cases showed postlingual hearing loss, with an average age of onset of 3.01 ± 1.86 years. The age of onset was unclear in the remaining 89 cases. In addition, 79 cases (22 prelingual cases and 57 postlingual cases) had clear histories of administration of aminoglycoside, with an average age of onset at 2.23 ± 1.71 years, and patients without a history of aminoglycoside use showed a significantly lower average age of onset of 0.75 ± 1.07 years (*P *< 0.001).

### GJB2 gene mutations

Sequence analysis of the *GJB2 *gene indicated that 51 patients carried two confirmed pathogenic mutations, and 1 patient had an R75W mutation, which has been reported to cause autosomal dominant syndromic deafness with palmoplantar keratoderma [44] (Table 1). Twenty-eight patients, including the 1 patient with autosomal dominant R75W mutation, were heterozygous for one pathogenic mutant allele. Four patients were heterozygous for one unclassified novel variant, the pathogenicity of which has not been determined (Table 1). In addition, 3 patients carried the heterozygous allele V37I, about which there is debate regarding whether it is a pathogenic mutation or a polymorphism [8,45-47]. Thus, 29.23% (83/284) of the unrelated families of deaf patients in typical areas in China had molecular defects in *GJB2*, and 18.31% (52/284) had confirmed molecular etiology of nonsyndromic hearing impairment (51 autosomal recessive and 1 autosomal dominant) in the *GJB2 *gene.

**Table 1:** Genotypes of patients with mutations in the *GJB2 *gene

**Allele 1**			**Allele 2**			
**Nucleotide Change**	**Consequence or amino acid change**	**Category**	**Nucleotide change**	**Consequence or amino acid change**	**Category**	**Number of patients**

c.235delC	Frameshift	Pathogenic	c.235delC	Frameshift	Pathogenic	31
c.235delC	Frameshift	Pathogenic	c.299_300delAT	Frameshift	Pathogenic	8
c.235delC	Frameshift	Pathogenic	c.176_191del16	Frameshift	Pathogenic	5
c.235delC	Frameshift	Pathogenic	c.257C>G	T86R TM2	Pathogenic	1
c.560_605ins46	Frameshift	Pathogenic	c.560_.605ins46	Frameshift	Pathogenic	1
c.299_300delAT	Frameshift	Pathogenic	c.176_191del16	Frameshift	Pathogenic	4
c.176_191del16	Frameshift	Pathogenic	c.176_191del16	Frameshift	Pathogenic	1
c.223C>T	R75W EC1 Autosomal dominant	^a^Pathogenic PPK	c.79G>A, c.341A>G	V27I, E114G	Polymorphism	1
c.235delC	Frameshift	Pathogenic	-			20
c.299_300delAT	Frameshift	Pathogenic	-			6
c.155_158delTCTG	Frameshift	Pathogenic	-			1
c.592G>A	^b^V198M TM4	Novel	c.79G>A, c.341A>G	V27I, E114G	Polymorphism	2
c.187G>T	^b^V63L EC1	Reported	-			1
c.458T>C	^b^V153AEC2	Novel	c.608T>C	I203T	Polymorphism	1
c.109G>A	^c^V37I, TM1	^c^See note	-			2
c.109G>A	^c^V37I	^c^See note	c.79G>A, c.341A>G	V27I, E114G	Polymorphism	1
c.79G>A, c.341A>G	V27I, E114G IC2	Polymorphism	-			42
c.79G>A, c.341A>G	V27I, E114G	Polymorphism	c.79G>A, c.341A>G	V27I, E114G	Polymorphism	2
c.341A>G	E114G	Polymorphism	-			1
c.79G>A	V27I TM1	Polymorphism	-			8
c.79G>A	V27I	Polymorphism	c.79G>A	V27I	Polymorphism	1

TM, transmembrane domain; EC, extracellular domain; IC, intracellular domain.

Five frameshift (235delC, 299_300delAT, 176_191del16, 560_605ins46, and 155_158delTCTG) and two missense (T86R and R75W) pathogenic mutations were found in this cohort (Table 1). The most prevalent mutation in this patient cohort was 235delC, which has also been reported to be the most prevalent mutation in other Asian populations [6,46]. Thirty-one patients were homozygous for 235delC mutation, 14 were compound heterozygous with another pathogenic mutation, and 20 were heterozygous for 235delC mutation (Table 1). Four novel alterations were identified, specifically, a frameshift pathogenic 155_158delTCTG mutation and three unclassified missense variants, V198M, V63L, and V153A (Tables 1). Overall, 134 mutant alleles (including the unclassified missense variants but excluding the V37I variant) were identified in 83 unrelated patients. 235delC alone accounted for 71.64% (96/134) of the total mutant alleles. Two mutations, 235delC and 299delAT, accounted for 85.07% (114/134) of the *GJB2 *mutations in our patients, 91% in another Chinese population [47], and 97% in a Taiwanese population [48]. These detection rates were higher among all the studies on the Asian deaf populations to date [6,10,45,46,48]. The V37I variant was considered a pathogenic mutation in Japanese studies, but it was not found in any of the Korean control or patient populations reported previously [6,10,46]. The frequency of V37I in our deaf population was lower than that in our control group (P < 0.05). T123N is an unclassified variant, which was counted as a mutation in a previous Japanese study but as a polymorphism in another study in Taiwan [10,45]. We found three T123N alleles in our control subjects but none in the patient group.

No variations in the *GJB2 *gene mutation spectra were found among the different ethnicities of Chinese patients in our study, with 235delC being the most common mutation in all ethnic groups. The 299_300delAT mutation was found in 15 Han, 1 Mon, and 1 Hui patient. The deleterious 560_605ins46 mutation was found in 1 Man patient. The 176_191del16 mutation was detected in 8 Han and 1 Mon patient, and 155_158 delTCTG was detected in 1 Man patient. Four of 7 Man patients (57%) and about 30% of patients from all other races [27.98% (68/243) of Han, 32.3% (10/31) of Mon, and 33.3% (1/3) of Hui] carried *GJB2 *mutations. No significant differences in *GJB2 *detection rate were found among these four ethnic groups (χ^2 ^= 2.4893, *P *= 0.4772).

We analyzed the *GJB2 *gene from 200 control subjects with normal hearing and found three types of deleterious mutation, 235delC, 299_300delAT, and 139G>T(E47X), carried by 7 subjects in the heterozygous state. This suggested a *GJB2 *mutation carrier rate of about 3.5% (7/200) in the general population. Meanwhile, the carrier rates of *GJB2 *mutation in Korea, Japan, Taiwan, among Ashkenazi Jews, and in the Midwestern United States were reported to be 2%, 2.08%, 2.55%, 4.76%, and 3.01%, respectively [5,6,45,46,49].

None of our patients heterozygous for one *GJB2 *mutant allele or the controls with normal hearing carried the IVS1+1G>A mutation or variant in exon1 and basal promoter of *GJB2*.

### Mutations in GJB6

None of our patients heterozygous for one *GJB2 *mutant allele or the controls with normal hearing had the known 309-kb deletion or other variant in the *GJB6 *gene.

### Mutations in mtDNA 12S rRNA and tRNA^ser(UCN)^

Five patients were found to carry the 1555A>G mutation, and 4 patients carried the 1095T>C mutation in the *mtDNA 12S rRNA *gene. Two patients were detected carrying the 7444G>A mutation in the *mtDNA tRNA*^*ser*(*UCN*) ^gene. All of the above 11 patients had a clear history of aminoglycoside use. None of the remaining 68 patients with history of aminoglycoside use had mutations in *12S rRNA *or *tRNA*^*ser*(*UCN*) ^in the mitochondrial genome. One of the 2 patients with 7444G>A mutation was also homozygous for the *SLC26A4 *IVS7-2A>G mutation and was further verified to have EVA by temporal CT scan. Thus, this patient may be only a 7444G>A carrier, with defects in *SLC26A4 *being the main cause of hearing loss. Two of the 200 control subjects were found to carry the *mtDNA 12S rRNA *1095T>C mutation, giving a carrier rate of 1% (2/200). Statistical analysis showed no significant difference in the incidence of the 1095T>C mutation between the patient and control groups. No other mutations were detected in the mitochondrial genome in the controls. All the mutations found in the mitochondrial genome were homogeneous.

### Mutations in SLC26A4

Sequence analysis of the *SLC26A4 *gene in these 227 patients with hearing impairment identified 28 patients with two confirmed pathogenic mutations (Table 2) and one compound heterozygote for two unclassified variants, Y375C and R470H, which are most likely pathogenic. Twenty-one patients carried one *SLC26A4 *mutant allele, and 2 patients carried novel unclassified missense variants, I491T and L597S, respectively, which are probably pathogenic due to the changes in evolutionarily conserved amino acids. Two patients carried V659L, including 1 who was verified to have EVA by CT scan. Wang *et al*. reported the pathogenicity of V659L in Chinese EVA patients [25]. Two unclassified heterozygous missense variants were found, I235V and T67S. The 2 patients carrying these single conserved amino acid changes had normal vestibular aqueducts. These two missense variants are probably benign, or these patients were only carriers of the mutation and their hearing impairment had other etiologies. One patient with normal results on temporal CT scan carried a novel variant, IVS12-6insT, in the heterozygous state. Analysis using the program NNSPLICE available at  did not predict gain or loss of a splice site with this variant, and it was therefore also considered benign. Thus, mutations in *SLC26A4 *were identified in 18.66% (53/284) of patients with hearing impairment in typical areas of China, 29 with two mutant alleles and 24 with one mutant allele.

**Table 2:** Genotypes of *SLC26A4 *gene-related hearing impairment in typical Chinese areas

**Allele 1**			**Allele 2**			**Number of patients**	
**Nucleotide Change**	**Amino acid change**	**Category**	**Nucleotide change**	**Amino acid change**	**Category**		

c.IVS7-2A>G	aberrant splicing	Pathogenic	c.IVS7-2A>G	Aberrant splicing	Pathogenic	16	EVA
c.2168A>G	H723R	Pathogenic	c.2168A>G	H723R	Pathogenic	1	EVA
c.1174A>T	N392Y	Pathogenic	c.1174A>T	N392Y	Pathogenic	1	EVA
c.IVS7-2A>G	aberrant splicing	Pathogenic	c.230A>T	K77I	Pathogenic	1	EVA
c.IVS7-2A>G	aberrant splicing	Pathogenic	c.1229C>T	^b^T410M	Pathogenic	1	EVA
c.IVS7-2A>G	aberrant splicing	Pathogenic	c.1975G>C	^b^V659L	Pathogenic	1	EVA
c.IVS7-2A>G	aberrant splicing	Pathogenic	c.2168A>G	H723R	Pathogenic	3	EVA
c.2168A>G	H723R	Pathogenic	c.109G>T	E37X, nonsense mutation	Pathogenic	1	EVA
c.2168A>G	H723R	Pathogenic	c.1229C>T	^b^T410M	Pathogenic	1	EVA
c.2168A>G	H723R	Pathogenic	c.2167C>G	H723D	Unclassified variant	1	EVA
c.1173C>A	S391R	Pathogenic	c.1229C>T	^b^T410M	Pathogenic	1	EVA
c.1124A>G	Y375C	Unclassified variant	c.1409G>A	R470H	Unclassified variant	1	Vestibular and cochlear malformation
c.1472T>C	I491T	Unclassified variant				1	EVA and Mondini
c.IVS7-2A>G	aberrant splicing	Pathogenic				8	EVA
c.2168A>G	H723R	Pathogenic				1	EVA
c.IVS7-2A>G	aberrant splicing	Pathogenic	c.1905G>A	E635E	Silent variant	1	ND
c.1174A>T	N392Y	Pathogenic				1	ND
c.IVS7-2A>G	aberrant splicing	Pathogenic				8	nl
c.2168A>G	H723R	Pathogenic				2	nl
c.1790T>C	L597S	Unclassified variant				1	nl
c.1975G>C	^b^V659L	Pathogenic				1	nl
c.757A>G	I253V	Unclassified variant				1	nl
c.200C>G	T67S	Unclassified variant				1	nl
c.IVS12-6i nsT	Intron insertion	Unclassified variant				1	nl
c.225C>G	L75L	Silent variant				1	ND
c.678T>C	A226A	Silent variant				1	nl
c.1905G>A	E635E	Silent variant				1	nl

nl, normal; EVA, enlarged vestibular aqueduct; ND, not determined; NA, not available; IVS7, intravening sequence 7 (intron 7); IVS12, intravening sequence 12 (intron 12).

A total of seven different pathogenic mutations (IVS7-2A>G, E37X, K77I, S391R, N392Y, T410M, H723R) and five novel, probably pathogenic variants (Y375C, R470H, I491T, L597S, and H723D) were found. The E37X mutation that results in a premature stop codon and a truncated protein less than 5% of the normal length is predicted to be deleterious. The H723D mutation is caused by nucleotide substitution, c.2167C>G, which was predicted to be deleterious as a milder change at the same amino acid residue, H723R, was shown to be the most common pathogenic mutation in Japanese subjects. Other missense mutations, K77I, S391R, N392Y, T410M, and H723R, have been reported in patients with hearing loss [24,25,50]. Y375C, R470H, I491T, L597S, and H723D were considered pathogenic, as they are located in an evolutionarily conserved region. The substituted amino acids are structurally and functionally different from those in the wild-type sequence, and Y375C, R470H, I491T, and H723D have been found in patients with EVA or other forms of inner ear malformation and were not found in our normal controls.

The most common mutation in our patient cohort was the aberrant splice-site alteration, IVS7-2A>G, for which 16 patients were homozygous, 4 were compound heterozygous, and 17 were heterozygous. The IVS7-2A>G mutation accounted for 64.63% (53/82, counting only the definite pathogenic and most likely pathogenic variants) of all *SLC26A4 *mutant alleles in this population (Table 2).

Three novel silent variants were identified in the patients, c.1905C>G (E635E), c.678T>C (A226A), and c.225C>G (L75L), which were not detected in the control group.

To determine the carrier frequency in the general population, *SLC26A4 *exons 2-21 of 200 individuals with normal hearing were analyzed by DHPLC. Four IVS7-2A>G heterozygotes and one silent variant, 2217A>G (Q739Q), were found. The carrier rate of the *SLC26A4 *mutation in China was estimated to be about 2%. Polymorphisms in the *SLC26A4 *gene appear to be rare in the general population in comparison to those in the *GJB2 *gene.

### CT scan

Temporal CT scan revealed EVA and/or other inner ear malformation in 39 patients. Twenty-eight patients had EVA and two pathogenic mutant alleles, consistent with an autosomal recessive disorder caused by biallelic loss of function of pendrin protein. One female patient carrying two novel missense variants, Y375C and R470H, had a common cystic cavity of the cochlea and vestibule without EVA. One male patient carrying a novel I491T variant had enlarged vestibular aqueducts with Mondini dysplasia. Eight patients with one mutant IVS7-2A>G allele had EVA. One patient with one mutant 2168A>G allele had EVA. CT scan results of 3 patients carrying heterozygous IVS7-2A>G, N392Y, and a polymorphism (L75L), respectively, were not available (Table 2). Temporal CT scan results were normal in the remaining patients. Testing of the two most frequent mutations, IVS7-2A>G and H723R, identified 89.74% of patients with EVA or inner ear malformation in this cohort.

### Thyroid ultrasound and thyroid hormone assays

Thyroid ultrasound was performed to determine the presence or absence of goiter. None of the patients with *SLC26A4 *mutations or variants showed the presence of goiter. Only 1 patient with EVA showed cystoid changes in the thyroid on ultrasound scan, whereas no changes were observed in thyroid hormone levels. Thyroid hormone assays showed that total T3 was slightly elevated in 2 patients, but this was of no clinical significance, according to endocrinologists from Chinese PLA General Hospital.

### Mutations in GJB3

Sequence analysis of the *GJB3 *gene identified five heterozygous variants in 44 patients: 24_49ins26bp (GCCATGGACTGGAAGACACTCCAGGC), 87C>T (F29F), 250A>G (V84I), 357C>T (N119N), and 497A>G (N166S) (Table 3). Both 87C>T and 357C>T are silent variants. Two patients were heterozygous for 250A>G (V84I). To clarify the pathogenicity of the V84I variant, we performed a control study in a group of 200 individuals with normal hearing. The frequency of V84I in the deaf population was not significantly different from that in the controls, but it was shown to be a *GJB3 *polymorphism in the Chinese population. One patient was heterozygous for 497A>G, which results in replacement of asparagine with serine at position 166 of Cx31. The patient carrying N166S mutation in one allele carried *GJB2 *235delC mutation in the other allele. The 24_49ins26bp variant is a novel frameshift, which results in a premature stop codon and a truncated Cx31 protein. In addition, 24_49ins26bp and N166S were detected only in patients with hearing impairment and not in the controls, and they are very likely to be deleterious mutations. Only 2 patients with *GJB3 *mutation were found in this cohort.

**Table 3:** Genotypes of patients and controls with variants in *GJB3 *gene

**Allele 1**			**Allele 2**			**Domain**	**Number of patients**^d^	**Number of controls**
**Nucleotide Change**	**Consequence or amino acid change**	**Category**	**Nucleotide change**	**Consequence or amino acid change**	**Category**			

c.24_49ins26bp	Frameshift	Novel pathogenic	-			IC1	1	
c.497A>G	N166S	Novel pathogenic	-			EC2	1	
c.580G>A	A194T	Unclassified	-			TM4		2
c.250A>G	V84I	Polymorphism	-			TM2	2	
c.250A>G	V84I	Polymorphism	c.250A>G	V84I	Polymorphism			1
c.357C>T	N119N	Polymorphism	-			IC2	39	38
c.357C>T	N119N	Polymorphism	c.357C>T	N119N	Polymorphism			2
c.327C>T	H109H	Novel Polymorphism	-			IC2		1
c.87C>T	F29F	Polymorphism	-			TM1	1	2

TM, transmembrane domain; EC, extracellular domain; IC, intracellular domain.

Five types of *GJB3 *variant were detected in the control group: 357C>T (N119N), 87C>T (F29F), 327C>T (H109H), 250A>G (V84I), and 580G>A (A194T). One control subject was homozygous for 250A>G (V84I). 327C>T is a silent variant. The variant 580G>A was predicted to replace the hydrophobic alanine at position 194 of Cx31 with a hydrophilic threonine (A194T). This variant was first found in 2 patients from China with autosomal dominant hearing loss and was considered to be a genetic cause in these two cases [51]. We regard A194T as an unclassified variant because it was not detected in any of our patients. Long-term follow-up is necessary in the 2 controls with A194T mutation to determine whether their hearing level will show any impairment in future.

## Discussion

### GJB2 gene

Previous reports suggested that the prevalence of *GJB2 *mutations varies among different ethnic groups. The most common mutation in Caucasians, 35delG, was not found in our patients. Instead, 235delC accounted for 71.64% of *GJB2 *mutant alleles in our cohort. This is mutation is detected at the highest rates among Asian populations, with incidences of approximately 41% and 57% in two Japanese reports, 67% in one Taiwanese study, and 73% in one Korean study [6,10,45,46,48]. The Chinese population is made up of six major ethnicities: Han, Man, Mon, Hui, Zhuang, and Miao. The majority are Han (91.6%), and this was also the predominant ethnicity in the study population (85.56%). No significant differences in *GJB2 *mutation spectra were found among different ethnicities in the Chinese population, although the numbers in the non-Han populations were too small to allow final conclusions to be reached in our study.

The missense mutation T86R was found in 1 patient who was also compound heterozygous for 235delC mutation. Although this mutation is not listed in the *GJB2 *mutation database website , it had been reported in 3 Japanese patients [10]. The 15-year-old Chinese female patient with R75W mutation developed thickening and peeling of the skin at medial and lateral sides of both hands and feet at 1 year of age. Pure-tone audiometry testing showed that her father had moderate high-frequency hearing loss, whereas her mother had normal hearing. Her father and mother did not have similar skin problems. GJB2 sequencing indicated that neither of her parents carried the R75W mutation. Therefore, R75W was a *de novo *mutation in this subject. This mutation has been reported previously in association with autosomal dominant deafness and palmoplantar keratoderma [44]. Three missense variants, V63L, V153A, and V198M, likely contribute to the pathogenesis of deafness, because they were detected only in the patient group and not in the control group, and they are evolutionarily conserved in Xenopus, mouse, rat, sheep, orangutan, and human. These mutations were heterozygous in 4 unrelated patients who carried only one mutant allele. It is not clear if they represent autosomal dominant mutations or are autosomal recessive with an as-yet unidentified second mutant allele in either the same gene (deep in introns or untranslated regions) or in different genes (digenic synergistic heterozygous mutations)[16,52]. Alternatively, these patients may simply be coincidental carriers whose deafness is caused by non-genetic environmental factors.

In our study population, 51 patients had two confirmed pathogenic mutations, plus the patient carrying the dominant R75W, and deafness in 18.31% (52/284) of our patients was due to mutations in *GJB2*. The percentage of *GJB2*-related hearing loss in other studies was 5.9-7% in Taiwan, 4.8% in Korea, 10.3% in the US, 13.5% in Australia, and 14.3% in Germany [6,8,9,45,48,53]. A significant proportion of patients with *GJB2 *mutations had only one mutant allele. Carriers of a single mutation in the *GJB2 *gene show evidence of reduced hair cell function [54]. Thus, it is possible that these carriers are more likely than are non-carriers to develop hearing impairment in the presence of other genetic defects or environmental factors. In addition to the common *GJB6 *309-kb deletion, *GJB2 *IVS1+1G>A is another mutant DFNB1 allele. Tóth *et al*. reported that 23.4% of Hungarian *GJB2*-heterozygous patients carried the splice-site mutation IVS1+1G>A in the 5'UTR region of *GJB2 *[55]. In addition, *GJB2 *mutations may act synergistically in the presence of *mtDNA *1555A>G mutation with aminoglycoside-induced ototoxicity [56]. Deletions in the *GJB6 *gene, the IVS1+1G>A mutation, or variants in exon1 and the basal promoter of *GJB2 *were not detected in any of the patients in the present study.

### SLC26A4 gene

*SLC26A4 *gene mutations were detected in nearly 20% of our nonsyndromic hearing impairment patients, with IVS7-2A>G being the most prevalent mutation. About 14% (39/284) of our cases were due to mutations in *SLC26A4*. The *SLC26A4 *gene is another common gene involved in deafness in typical areas in China. To identify Pendred syndrome in the EVA patients, we performed thyroid hormone testing and ultrasound scan of the thyroid to examine the function and structure of the thyroid instead of perchlorate discharge testing, a routine method used for examining thyroid function that is not available in most areas of China. Our results indicated that none of patients had Pendred syndrome. The discrepancy between our results and those of previous studies may be explained by differences in testing methods used; the age of the patients, as those undergoing thyroid ultrasound and thyroid hormone assays in this study (3 to 20, average 12.3 ± 2.7) may have been too young to show symptoms; and/or phenotypic diversity due to differences in genetic background.

It is interesting to note that the 10 patients with inner ear malformation carried one missense mutation only. Whether the missense mutation causes a dominant negative effect and/or specifies a different phenotype is not clear. It is possible that the second mutant allele has not yet been identified due to the location of mutations deep in introns or promoter regions that were not sequenced, intragenic exon deletions, or the involvement of mutations in genes other than *SLC26A4 *in the pathogenesis (*i.e*., digenic synergistic mutations).

The *SLC26A4 *mutation spectrum in typical areas in China is similar to that reported in the overall Chinese population but different from that in Japan. Research findings indicate a gradient shift of the most prevalent mutation from IVS7-2A>G to H723R from Chinese to Japanese, respectively, with both mutations being equally prevalent in the Korean population. This observation suggests that IVS7-2A>G and H723R mutations may be ancient mutations in China and Japan, respectively. A recent study by Albert *et al*. of 100 unrelated patients with EVA in European Caucasian subjects revealed a diverse mutation spectrum without prevalent mutations, and only 40 patients carried *SLC26A4 *mutations [24]. It is not clear why the mutations in *SLC26A4 *account for a much lower percentage of patients with EVA in Caucasian populations. Presumably, other genetic factors and environmental factors are involved in the pathogenesis of EVA in Caucasian populations.

We found no significant differences in the spectrum or prevalence of *GJB2 *and *SLC26A4 *between patients from Chifeng City and those from Nantong City.

### mtDNA 12S rRNA and mtDNA tRNA^ser(UCN)^

All 5 patients with 1555A>G mutation in the present study had a history of aminoglycoside use. Pedigree analysis showed maternally inherited traits, and these patients were diagnosed as having aminoglycoside-induced nonsyndromic hearing loss. We investigated the clinical and molecular characteristics of three of the four *mtDNA *1095T>C pedigrees. The extremely low penetrance of hearing loss in the Chinese families carrying the 1095T>C mutation strongly suggested that the 1095T>C mutation itself is not sufficient to produce the clinical phenotype. Therefore, other modifiers, including aminoglycosides, nuclear genes, and mitochondrial haplotypes, are necessary for the phenotypic manifestation of the 1095T>C mutation. Despite the presence of several highly evolutionarily conserved variants in protein-coding genes and the 16S rRNA gene [57], the extremely low penetrance of hearing loss with the 1095T>C mutation implies that the mitochondrial variants may not have a modifying role in phenotypic expression of the 1095T>C mutation in these Chinese families. However, the history of exposure to aminoglycosides in these 3 hearing-impaired subjects suggested that these agents were probably the cause of hearing loss. Two controls were also found to carry the 1095T>C mutation; they were advised to avoid use of aminoglycosides, and their hearing level is being followed closely.

The 7444G>A substitution has been described in deaf individuals with and without the 1555A>G mutation, but its pathogenicity has not been established [58]. Yao *et al*. considered 7444G>A to be a normal polymorphism [59]. The patient with *mtDNA *7444G>A mutation, who began suffering bilateral hearing impairment within 3 months after administration of streptomycin, had no relevant family history. We performed PCR amplification of fragments spanning the entire mitochondrial genome, and subsequent DNA sequence analysis in this patient revealed no variants in evolutionarily conserved regions in the mitochondrial genome. The molecular etiology of the patient carrying 7444G>A mutation remains to be identified.

### GJB3 gene

Richard *et al*. [60] identified three mutations in the Connexin31 gene (*GJB3*) in four families with erythrokeratodermia variabilis (EKV). Independently, Xia *et al*. [13] reported cloning of the human *GJB3 *gene on chromosome 1p33-p35 and found mutations in two small families with deafness. The observation that some carriers of *GJB3 *mutations showed a normal phenotype challenges the involvement of these mutations in dominant deafness. *GJB3 *has been shown to be related to early-onset autosomal recessive deafness. In the present study, the patient carrying N166S mutation in one allele was verified to carry *GJB2 *235delC mutation in the other. Direct physical interaction of Cx26 with Cx31 is supported by data showing that Cx26 and Cx31 have overlapping expression patterns in the cochlea. In addition, we identified the presence of heteromeric Cx26/Cx31 connexons by coimmunoprecipitation of mouse cochlear membrane proteins. Furthermore, by cotransfection of mCherry-tagged Cx26 and GFP-tagged Cx31 into human embryonic kidney (HEK)-293 cells, we demonstrated that the two connexins were able to co-assemble *in vitro *in the same junction plaque. The above data indicate that a genetic interaction between *GJB3 *and *GJB2 *can lead to hearing loss [61]. A diagnosis of digenic inherited *GJB2 *and *GJB3 *hearing loss was made in this patient. The frameshift mutation 24_49ins26bp (GCCATGGACTGGAAGACACTCCAGGC) generates a putative truncated protein of only 18 amino acids. The patient carrying *GJB3 *24_49ins26bp in our cohort had congenital symmetric hearing loss with no relevant family history. The severity of her hearing impairment was profound. Unfortunately, blood samples from her parents were not available for analysis. If one of the parents with normal hearing carries this mutation, the patient may only be a carrier. Alternatively, if neither of the parents with normal hearing carries this mutation, the 24_49ins26bp mutation in the patient may have arisen *de novo *and may be the genetic cause or at least one of the factors responsible for her phenotype.

Taken together, approximately 47.89% (83 + 53/284) of patients with NSHI in typical Chinese areas had molecular defects in the *GJB2 *or *SLC26A4 *gene, whereas about 33.1% and 3.5% of European patients with NSHI carried mutations in *GJB2 *and *SLC26A4*, respectively, with a total of 36.6% in a patient cohort of 142 sib pairs [30]. *MtDNA *1555A>G mutation accounted for the etiology in 1.76% (5/284) of the patients with hearing loss. Ten patients with a family history of hearing loss showed mutations in *GJB2, GJB3, GJB6, SLC26A4, mtDNA 12S rRNA*, or *mtDNA tRNA*^*ser*(*UCN*) ^in our study population. The etiologies of these 10 patients are most likely genetic, although no mutations in common hearing loss genes were found. If the 4 patients with 1095T>C in *mtDNA 12SrRNA *and 1 patient carrying *GJB3 *24_49ins26 were all included, hearing loss in 54.93% (156/284) of our Chinese patients was related to genetic factors.

This is the first comprehensive study of the molecular etiology of nonsyndromic hearing impairment in mainland China. *GJB2 *and *SLC26A4 *are the two most common etiologies for deafness in the Chinese population. A preliminary investigation of the mutation spectrum and prevalence of *GJB2 *and *SLC26A4 *between typical areas from northern and southern China was performed in this study, and no significant differences were found.

## Conclusion

In this study, a total of 54.93% of Chinese patients with hearing impairment showed evidence of genetic involvement either based on genetic screening or family history, and 18.31%, 13.73%, and 1.76% of the patients were determined to have inherited hearing impairment caused by *GJB2*, *SLC26A4*, and *mtDNA *1555A>G mutations. Mutations in *GJB3*, *GJB6*, and *mtDNA tRNA*^*ser*(*UCN*) ^are not common. Screening for *GJB2*, *SLC26A4*, and *12S rRNA *should be considered the first step in genetic testing of deaf Chinese patients. Furthermore, the molecular defects of about 66% of the patients with nonsyndromic hearing impairment in China remain to be identified.

## Competing interests

The authors declare that they have no competing interests.

## Authors' contributions

YoYu, YiYo, and DH carried out the molecular genetic studies and participated in sequence alignment. YoYu drafted the manuscript. YW and QW carried out temporal CT scan and thyroid hormone assays. JC, FY, and DK participated in sequence alignment and performed the statistical analyses. HY and DH participated in the design of the study. PD conceived the study, participated in its design and coordination, and helped draft the manuscript. All authors have read and approved the final manuscript.
